# Kinematic Gait Impairments in Children with Clubfeet Treated by the Ponseti Method: A Systematic Review and Meta-Analysis

**DOI:** 10.3390/children10050785

**Published:** 2023-04-26

**Authors:** Lianne Grin, Lisa van Oorschot, Benedicte Vanwanseele, Saskia D. N. Wijnands, H. J. J. (Cojanne) Kars, Arnold T. Besselaar, M. C. (Marieke) van der Steen

**Affiliations:** 1 Department of Health Innovations and Technology, Fontys University of Applied Sciences, Dominee Theodoor Fliednerstraat 2, 5361 BN Eindhoven, The Netherlands; 2 Department of Movement Sciences, Katholieke Universiteit Leuven, Tervuursevest 101, 3001 Heverlee, Belgium; 3 Department of Orthopaedic Surgery & Trauma, Máxima Medical Center, 5600 PD Eindhoven, The Netherlands; 4 Department of Orthopaedic Surgery & Trauma, Catharina Hospital Eindhoven, 5602 ZA Eindhoven, The Netherlands

**Keywords:** congenital talipes equinovarus, gait analysis, functional evaluation, relapse, multi-segment foot model

## Abstract

Background: Being aware of possible gait impairments in Ponseti-treated clubfoot children might be useful for optimizing initial and additional treatment. Therefore, this systematic review and meta-analysis aimed to identify kinematic gait abnormalities in children with clubfoot treated with the Ponseti method (with and without relapse). Methods: A systematic search was conducted. Studies comparing kinematic gait parameters of Ponseti-treated clubfoot children to healthy controls were included. Meta-analyses and qualitative analyses were conducted on the extracted data. Results: Twenty studies were identified. Twelve of the 153 reported kinematic outcome measures could be included in the meta-analysis. Plantarflexion at push-off, maximum ankle dorsiflexion during the swing, maximal plantarflexion, and ankle range of motion was significantly lower in Ponseti-treated clubfoot children. Ponseti-treated clubfoot children showed more internal foot progression. Qualitative analysis revealed 51 parameters in which pre-treatment relapse clubfeet deviated from healthy controls. Conclusions: Ponseti-treated clubfoot children showed several kinematic gait differences from healthy controls. In future studies, homogeneity in measured variables and study population and implementation of multi-segmental foot models will aid in comparing studies and understanding clubfoot complexity and treatment outcomes. The question remains as to what functional problems gait impairments lead to and whether additional treatment could address these problems.

## 1. Introduction

Worldwide approximately 100,000 children are born with unilateral or bilateral clubfoot (*talipes equinovarus*) yearly [1,2,3]. This deformity of the foot involves the *equinus*, *varus*, *cavus*, and *adductus* [4]. Left untreated, clubfoot leads to deformity, functional disability, and pain [5]. The treatment of this condition aims to achieve a normal-appearing, functional, and painless foot [6]. Nowadays, the Ponseti method is the gold standard for the initial treatment [5,7]. The Ponseti method consists of serial manipulations and casting combined with an Achilles tenotomy. The casting phase is followed by a brace period up to the age of 4 years to prevent relapses during early life [4,5].

Despite the effects of good initial treatment, reported relapse percentages following treatment with the Ponseti method range from 1.9% up to 67.3% [8,9,10]. The prevention and treatment of a relapse clubfoot are one of the great challenges in clubfoot care. Strict adherence to the Ponseti method, good brace compliance, and frequent clinical follow-up visits are important aspects of preventing relapse [11]. Although a clear definition is lacking, the common consensus is that a relapsed clubfoot requires additional treatment following initial correction [8]. This treatment may vary from repeated Ponseti casting to Tibialis Anterior Tendon Transfer (TATT) and a la carte salvage procedures such as anterior distal tibial epiphysiodesis [11,12].

Besides the occurrence of relapse also, the functional status of the patient is of interest. Functioning in children can be captured using the International Classification of Functioning, Disability, and Health for Children and Youth (ICF-CY) [13]. The ICF-CY contains three main aspects which affect a child’s functioning: (1) body structures and function, (2) activities, and (3) participation. Although these aspects together are considered to give a complete overview of the functioning of children, most research on outcomes of treatment in clubfoot patients focuses on body structures and function [13,14]. Extensive 3D gait analysis is a frequently applied tool to evaluate body structures and function, as part of the ICF, in the treatment outcomes [15] and to detect early signs of relapse [11]. 

With 3D gait analyses, objective kinematic and kinetic parameters of clubfoot patients can be derived [16,17,18]. Ponseti-treated clubfoot patients previously showed impairments in kinetic outcome measures, such as ankle plantar flexor moment and ankle power [19]. These kinetic outcomes depend on a child’s movement pattern, including joint angles. Hence, in order to establish whether a fully functional foot is achieved after initial treatment with the Ponseti method, kinematic parameters are also of interest. In the past few years, an increasing number of studies regarding gait kinematics in children with Ponseti-treated clubfeet have been published. A systematic overview of the reported gait deviations in various clubfoot populations provides insights into the functional outcome of the Ponseti method. Being aware of possible gait impairment is potentially useful for optimizing the Ponseti method, the detection of relapse clubfoot, and developing additional (physio)therapy or surgical treatment [20]. Therefore, this systematic review and meta-analysis aimed to identify kinematic gait abnormalities in children with clubfoot treated with the Ponseti method (with and without relapse).

## 2. Materials and Methods

### 2.1. Protocol and Registration

The protocol for this review was registered in the prospective international register of systematic reviews: PROSPERO number CRD42022375837. The Preferred Reporting Items for Systematic Reviews and Meta-Analysis (PRIMA) guidelines 2020 were applied while conducting and reporting this systematic review [21,22,23].

### 2.2. Eligibility Criteria 

Articles should be published in peer-reviewed journals in English, Dutch, or German. Studies comparing kinematic gait parameters of children with clubfoot treated with Ponseti to healthy controls were included. Studies describing the result of 3D gait analyses as an outcome of the Ponseti treatment as well as 3D gait analyses pre-relapse treatment, were considered. A minimum of 5 participants per group was set, and a 3D recording system for gait analysis was required. Cross-sectional, retrospective, and prospective follow-up studies were eligible, and book chapters, conference abstracts, and reviews were excluded. Furthermore, studies using only pedobarography or electromyography to determine gait parameters were excluded.

### 2.3. Literature Search

A literature search was conducted in the Embase, Medline Ovid, Web of Science, Scopus, Cochrane, Cinahl Ebsco, and Google Scholar databases by an experienced information specialist on 3 October 2022. Search terms included synonyms of clubfoot, gait analysis, and specific clubfoot treatments, such as Ponseti (Appendix A). Duplicates were removed. In addition, reference lists of related articles were checked for additional relevant references. 

### 2.4. Study Selection Procedure

A systematical selection of articles was made independently by two of the three researchers involved in this phase (MS, LO, and LG). Titles and abstracts of the obtained articles were screened on relevance with a focus on gait analysis in children with club feet. After this first selection, full texts were examined on content and relevance by two researchers (MS, LO, and LG). The absence of consensus on eligibility was resolved by a discussion between the researchers. 

### 2.5. Data Extraction

Data were extracted by one researcher (LO or LG) with the use of a data extraction form. The accuracy of the data extraction was verified by a second researcher (LG or MS). Study characteristics and kinematic outcome measures were extracted with respect to the segment (foot, ankle, etc.), the moment during the gait cycle (stance, gait, terminal stance, etc.), the actual outcome, and whether there was a significant difference between clubfoot patients and healthy controls and the type of clubfoot population (clubfoot without relapse, clubfoot with relapse for which additional treatment was planned or overcorrected clubfoot). In case of lack of clarity, authors were contacted via email for additional information. 

### 2.6. Risk of Bias Assessment

Individual examination of the risk of bias was performed for each study separately and performed by two researchers (MR and BV or MS and LG). The Dutch checklist for prognosis (Cochrane Netherlands) was applied with modifications to the items set to the relevance of the current study objectives (Appendix B). Items focused on the selection of participants, comparability of groups, description of groups, and a validated and blinded measurement of outcome. Items could be scored with ‘low risk’ (+), ‘high risk’ (−), or ‘unclear’ (?). The individual forms were compared and discussed for final consensus.

### 2.7. Data Synthesis and Analysis

Meta-analyses were performed for outcome measures that were reported with mean and standard deviation by at least three studies and gathered in the same clubfoot population (clubfoot without relapse, pre-treatment relapse, or overcorrected clubfoot). All meta-analyses were performed using Review Manager (RevMan 5.4.1) (Copenhagen, The Cochrane Collaboration, 2020). Kinematic outcome measures, which were presented separately for unilateral clubfoot and bilateral clubfoot, were merged using the RevMan Calculator and were considered as one group in this review and meta-analyses. The consistency of results was estimated with I^2^ statistics. In cases of no significant statistical heterogeneity, the fixed effects model was used. The random effects model was used in statistical heterogeneity cases (I^2^ > 50% and *p* < 0.05). If outcome measures were discussed in two or fewer studies, they were compared in a descriptive manner.

## 3. Results

### 3.1. Study Selection and Characteristics

**Figure 1:**
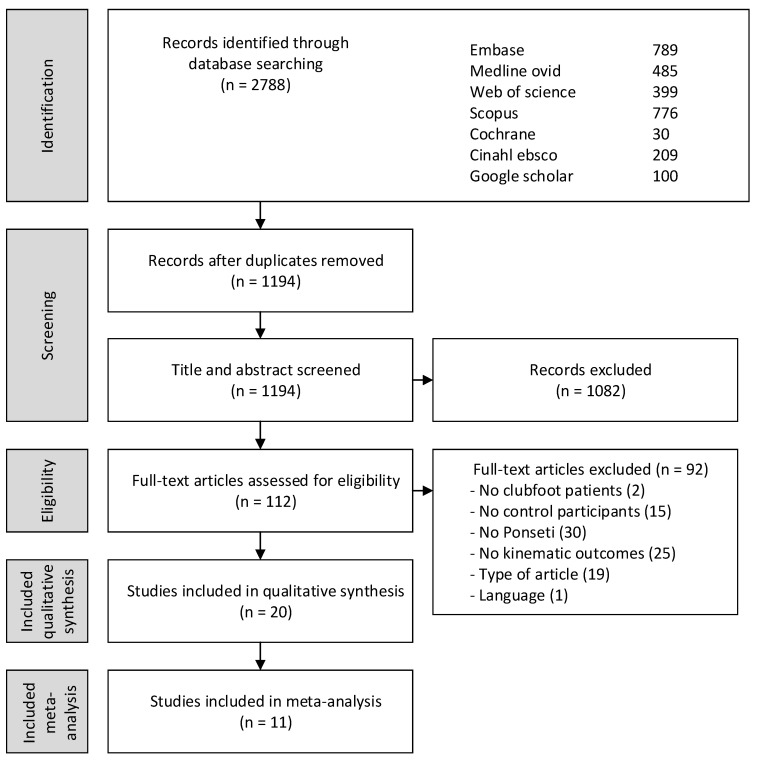
Flowchart selection procedure.

Initially, the search strategy provided 1194 unique articles. After screening articles for inclusion and exclusion criteria, 20 studies met the criteria [24,25,26,27,28,29,30,31,32,33,34,35,36,37,38,39,40,41,42,43]. Articles were mainly excluded since the described clubfoot cohort was not treated with the Ponseti method, and no kinematic outcomes were reported (Figure 1). 

Fifteen studies focused on kinematic outcomes after treatment with the Ponseti method [24,25,27,30,32,33,34,36,37,38,39,40,41,42,43], seven studies presented data from clubfoot patients prior to additional treatment for relapse [28,29,30,31,33,34,35], and one study described 3D gait analysis performed on overcorrected clubfoot [26]. Since the overcorrected clubfoot is a single specific group, the results of this study are presented in the Appendix C (Table A2). In 16 studies, children walked at a self-selected speed [24,26,27,28,29,30,31,33,34,35,36,38,40,41,42,43]. In the other four included studies, no information on walking speed was provided [25,32,37,39]. An overview of the study and participant characteristics of the included studies is shown in Table 1.

A large diversity of outcome measures was presented in the different studies (addressed in Section 3.3, Section 3.4 and Section 3.5). Twelve parameters described in eleven studies could be included in the meta-analyses. Lööf et al. (2016) made a clear distinction between unilateral clubfoot and bilateral clubfoot and compared them to the same group of healthy controls. This violates the assumptions of independence of observation that underpin the meta-analyses. Therefore, kinematic outcomes presented in Lööf et al. (2016) for uni- and bilateral clubfoot were merged using the RevMan Calculator and were considered as one group in this review and meta-analyses. 

### 3.2. Risk of Bias Assessment

The risk of bias assessment for each study separately showed the unclear or high risk of bias for one or more items (Appendix B, Table A1). This was mostly due to a lack of information or no information at all presented in the included articles

### 3.3. Meta-Analysis Clubfoot Treated with the Ponseti Method versus Controls

**Figure 2:**
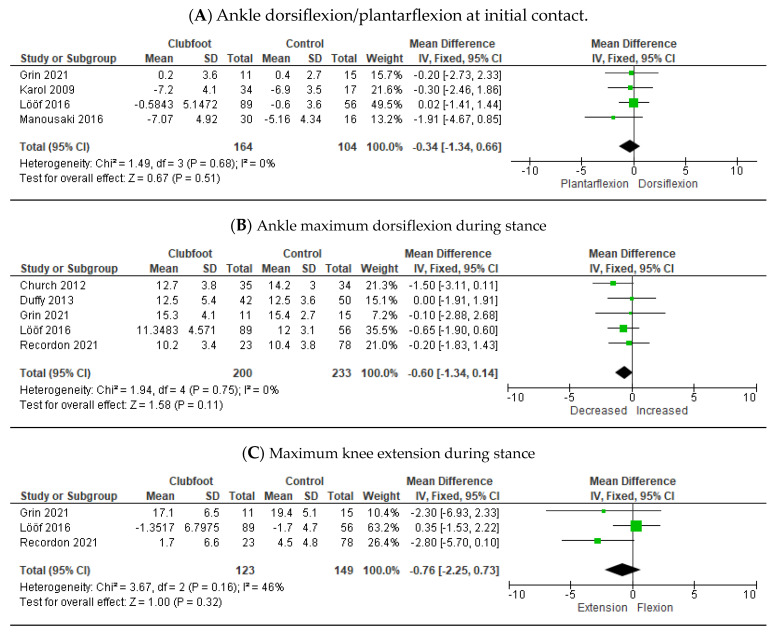

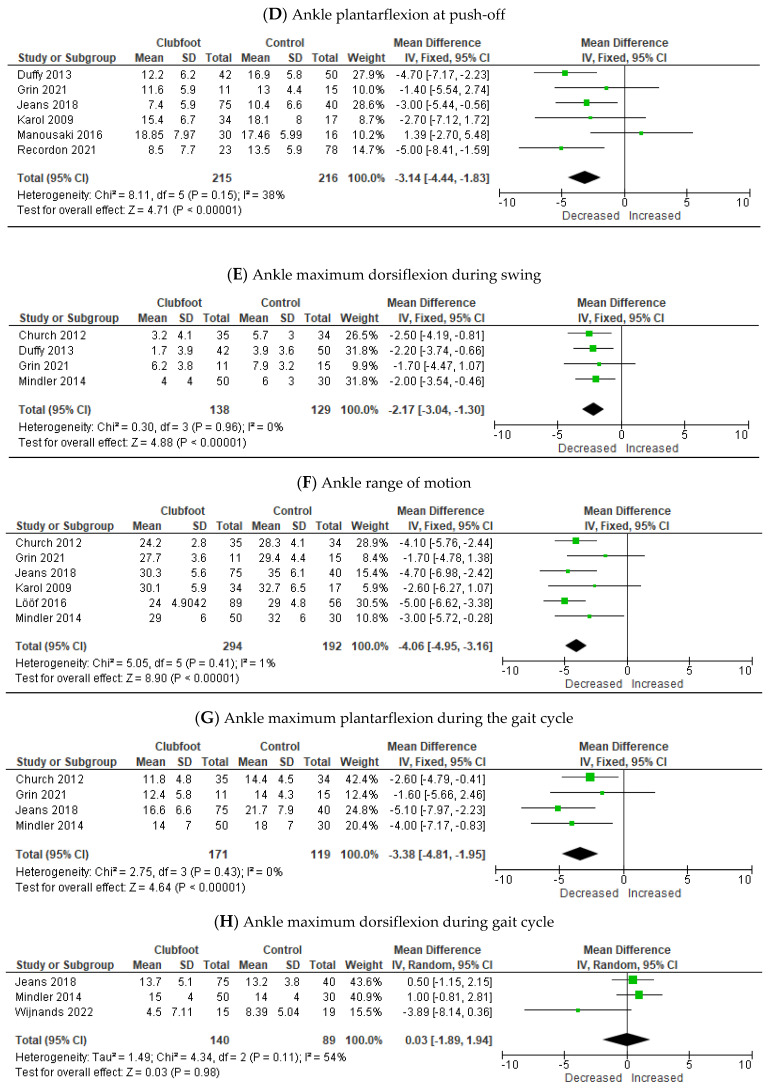
Meta-analysis parameters sagittal plane comparing clubfoot treated with the Ponseti method versus healthy controls [24,25,30,32,34,36,38,39,40,41].

A total of twelve outcome measures could be included in the meta-analyses. Eight of these measures involved the movements of the ankle and knee joints in the sagittal plane during different phases of the gait cycle. Results showed no overall significant differences between children with Ponseti-treated clubfeet and healthy controls at initial contact and during the stance phase (Figure 2A–C). 

At push-off, Ponseti-treated clubfeet showed a decreased plantarflexion [−3.14° (95% CI, −4.44–−1.83; *p* < 0. 001)] (Figure 2D). During the swing, maximum dorsiflexion in the ankle for Ponseti-treated clubfoot was significantly lower compared to healthy controls [−2.17° (95% CI, −3.04–−1.30; *p* < 0.001)] (Figure 2E). Over the whole gait cycle, Ponseti-treated clubfeet had a decreased range of motion in the ankle compared to healthy controls [−4.06° (95% CI, −4.95–−3.16; *p* < 0.001)] (Figure 2F) and a decreased maximal plantarflexion [−3.38° (95% CI, −4.81–−1.95; *p* < 0.001)] (Figure 2G). No overall significant difference was seen in maximum dorsiflexion (Figure 2H).

**Figure 3:**
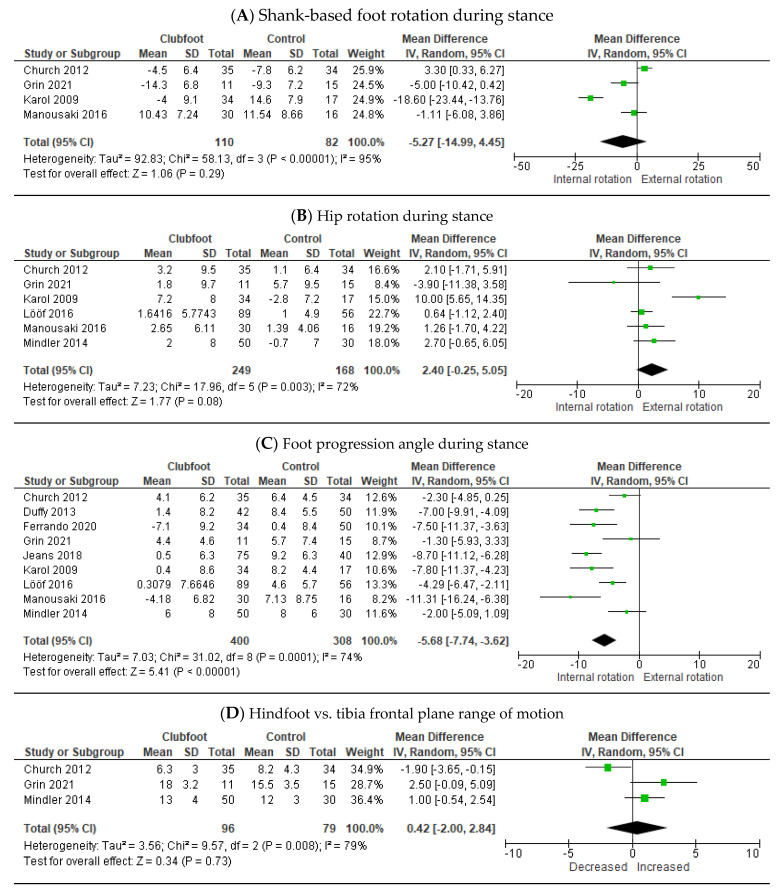
Meta-analysis parameters transversal (**A**–**C**) and frontal (**D**) plane comparing clubfoot treated with the Ponseti method versus healthy controls [24,25,27,30,36,38,39,40,41].

The four other included measures that could be included in the meta-analyses involved movements in the transversal plane and the frontal plane (Figure 3). 

No overall difference was seen in shank-based foot rotation (Figure 3A) and hip rotation (Figure 3B) during stance. Compared to healthy controls, children with Ponseti-treated clubfeet showed overall a more inward-oriented foot progression angle during stance [−5.68° (95% CI, −7.74–−3.62; *p* ≤ 0.001] (Figure 3C). Furthermore, no overall difference was seen in the frontal plane range of motion of the hindfoot in relation to the *tibia* (Figure 3D).

### 3.4. Qualitative Analysis Clubfoot Treated with the Ponseti Method versus Controls

An overview of outcome measures not eligible for inclusion (<3 articles or no standard deviation presented [43]) in the meta-analysis but reported in the different articles is displayed in Table 2 and Appendix D. 

When comparing children with clubfeet and healthy controls, no significant difference was found for 67 outcomes (Appendix D). A significant difference was found for nine outcome measures, and conflicting results were found for eight outcome measures (Table 2). The outcome measures with a significant difference between the groups and variables with contradicting results are described below.

#### 3.4.1. Stance Phase

From initial contact to mid-stance, no significant differences were reported. At mid-stance, one study mentioned a significantly smaller dorsiflexion in the ankle in Ponseti-treated clubfeet compared to the healthy controls [39], which is in conflict with another study where no significant difference was found [24]. Furthermore, Ponseti-treated clubfeet showed less forefoot plantarflexion in relation to the hindfoot compared to healthy controls [37]. During mid-stance, mean external hip rotation was increased in the clubfoot group, whereas maximum knee extension was decreased in this group compared to healthy controls [36]. Another study mentioned less maximum knee extension in children with Ponseti-treated clubfeet compared to healthy controls during the second half of the stance phase [38]. Subsequently, maximum plantarflexion in the ankle was decreased at a terminal stance in children with Ponseti-treated clubfeet compared to the healthy controls [40]. Furthermore, less external *tibial* torsion during stance was found in children with Ponseti-treated clubfoot compared to the healthy controls [25]. The foot progression angle during pre-swing was higher in the clubfoot group compared to healthy controls [37]. 

#### 3.4.2. Swing phase

During the swing phase, decreased maximum knee flexion and decreased dorsiflexion in the ankle were found in children with Ponseti-treated clubfeet compared to healthy controls [37,38], which is in conflict with another study where, although similar trend, no significant difference was found for both parameters [30].

#### 3.4.3. Gait Cycle

When considering the entire gait cycle, mean hip abduction was increased in children with Ponseti-treated clubfeet compared to controls [38], whereas a conflicting result was found looking at maximum external hip rotation [30,38]. In one study, Ponseti-treated clubfeet showed increased external hip rotation [38], whereas the other study showed no significant differences [30]. Furthermore, using a multi-segment foot model, several conflicting results regarding the range of motion (ROM) were observed in the different foot segments [30,38]. One study showed a decreased sagittal range of motion for the forefoot in relation to the hindfoot as well as in relation to the *tibia*, a decreased transversal range of motion for the forefoot in relation to the *tibia,* and an increased range of motion in the frontal plane for the forefoot in relation to the *tibia* in Ponseti treated clubfeet compared to healthy controls [38]. Another study showed no significant differences for the previously mentioned range of motions [30]. When looking at the total gait pattern using the Gait Deviation Index (GDI), children with Ponseti-treated clubfoot showed a decreased GDI score compared to healthy controls [36,39,43].

### 3.5. Qualitative Analysis Pre-Treatment Relapsed Clubfoot versus Controls

Despite a large number of kinematic outcome measures, there were no outcome measures eligible for inclusion (<3 articles or no standard deviation presented [28]) in the meta-analysis. An overview of all outcome measures that are reported in the different articles is displayed in Table 3 and Appendix E. 

Of the total of 106 outcome measures for 55 outcomes, no significant difference was found (Appendix E); for 32 outcome measures, a significant difference was found between children with pre-treatment relapsed clubfeet and healthy controls, and 19 outcome measures from different studies showed conflicting results (Table 3). The outcome measures with a significant difference between the groups and variables with contradicting results are described below.

#### 3.5.1. Multi-Segment Foot Model

Most significant differences between children with pre-treatment relapsed clubfeet and healthy controls are found at foot level, analyzed using a multi-segment foot model. These differences were present in all three planes and multiple phases of gait. In the sagittal plane, children with a relapse showed a significantly decreased forefoot plantarflexion in relation to the *tibia* at toe-off and increased forefoot dorsiflexion in relation to the *tibia* at 80% of the gait cycle [29]. In the frontal plane, children with a relapse showed increased forefoot supination in relation to the *tibia* at initial contact and in relation to the hindfoot at 80% of the gait [29]. Furthermore, increased hindfoot inversion in relation to the *tibia* was seen during the entire gait cycle [35]. In the transversal plane, children with a relapse walked with a more internally shank-based foot rotation [30], a smaller foot progression angle [29,35], and increased forefoot and hindfoot adduction during all phases of gait [28,29,30,35]. In relation to the *tibia,* increased forefoot adduction was found during initial contact [29], during stance [29,30], at 80% of the gait cycle [29], and over the full gait cycle [28]. Increased forefoot adduction in relation to the *tibia* was found at the toe-off [30] and over the full gait cycle [35]. For the hindfoot, increased adduction was found in relation to the *tibia* during the full gait cycle [28,35]. 

#### 3.5.2. Conventional Gait Model

When looking at the ankle, a decreased plantar flexion at the toe-off and a smaller sagittal range of motion is seen in children with a relapse [29,30]. Furthermore, children with a relapse showed less external knee rotation and more external hip rotation during stance [29,35]. During the swing, increased knee flexion and increased hip abduction were seen [35]. Additionally, when looking at the total gait pattern using several total gait scores, children with a relapse showed a deviated walking pattern compared to healthy controls [28,31,33].

#### 3.5.3. Conflicting Results

A close look at the conflicting results revealed that one of the nineteen conflicts is also a contradicting result. Two studies presented a decreased transversal range of motion for the hindfoot in relation to the *tibia* [29,30], while one other study showed an increased range of motion in children with relapsed clubfeet [28]. The eighteen remaining conflicting outcomes showed a difference in significance. However, no difference in the direction of deviation in joint angles was seen.

## 4. Discussion

This systematic review identified a total of 153 different kinematic outcome measures, presented in 20 studies on gait analyses in clubfeet patients treated with the Ponseti method with and without relapse compared to healthy controls. Twelve parameters could be included in a meta-analysis. These meta-analyses comparing Ponseti-treated clubfoot children without relapse to healthy controls showed overall significant differences in ankle plantarflexion at push-off and maximal ankle plantarflexion during the gait cycle, maximum ankle dorsiflexion during the swing, ankle range of motion, and the foot progression angle during stance. Furthermore, on 17 and 51 different kinematic outcomes, one or more studies reported deviating results in respectively clubfoot patients without relapse and pre-treatment relapsed clubfeet compared to healthy controls. 

Children with clubfoot have significantly decreased ankle plantar flexion angle at push-off, which is probably caused by a weakness or insufficiency of the plantar flexor muscles [36,45]. Smith et al. (2014), as well as Jeans et al. (2018), reported a decreased plantar flexor strength in children with Ponseti-treated clubfoot compared to healthy controls [37,41]. This finding is also in line with previous findings regarding decreased ankle power in children with clubfeet [19].

Significantly less maximum dorsiflexion during swing was seen in the Ponseti group, which can indicate a drop foot [38], and can consequently lead to insufficient floor clearance and forefoot landing [46]. Lack of dorsiflexion during the swing can lead to compensations which are mostly seen in an increased hip flexion to lift the foot [46]. Brierty et al. (2022) and Grin et al. (2021) found no significant difference in the hip flexion angle during the full gait cycle using statistical parametric mapping (SPM) [30,35]. However, the results of the meta-analysis on hip rotation did show, although not significant, a tendency for increased external hip rotation. Additionally, one study presented increased hip abduction in children with club feet [38]. Hip rotation and hip abduction are part of a circumduction movement that could also be used to compensate for a decreased foot clearance due to a lack of dorsiflexion. Furthermore, from a clinical point of view, more knee flexion during the initial swing and mid-swing could also be expected to compensate for less dorsiflexion. However, in the two studies that reported knee flexion during swing, a decreased maximum knee flexion was found [30,38]. 

In addition, it should be noted that three out of the four studies included in the meta-analysis that reported less maximum dorsiflexion during swing also included children with a *tibialis anterior tendon transfer* (TATT) as part of the Ponseti protocol in their study population [25,36,38]. This early TATT was previously associated postoperatively with impaired passive dorsiflexion in a randomized controlled trial comparing the Ponseti method with early TATT (without Ponseti casting) [47]. However, it needs to be questioned whether this small (approximately 2 degrees) but significant difference in maximum dorsiflexion during gait will lead to functional problems in the clubfoot group and, as such, should be addressed in additional treatment. 

As a result of a significantly decreased maximum ankle plantar flexion angle over the full gait cycle and a tendency to a decreased maximum ankle dorsiflexion angle during stance, children with a clubfoot showed a significantly decreased ankle range of motion in the sagittal plane. A limited range of motion can negatively affect a child’s second ankle rocker and the ability to push off, which are needed for a normal translation of the center of mass during stance. From a clinical point of view, either decreased plantar flexion or decreased dorsiflexion can be treated clinically; however, it requires differentiation in the treatment approach. 

A more internally rotated foot progression angle may lead to more compensatory external hip rotation in the transversal plane [48]. Correspondingly, a significantly more internally rotated foot progression and a tendency of increased external hip rotation during stance were found in clubfoot children compared to healthy controls. Additionally, one study reported an increased external hip rotation during mid-stance [36]. However, another study looked specifically at external hip rotation at initial contact and did not find a significant difference between clubfoot children and healthy control children [40]. Further, any torsional or foot deformations contributing to in-toeing could be compensated by external hip rotation during gait. These compensatory mechanisms highlight the importance of considering the entire kinematic chain for the clinical evaluation of gait analysis [49].

The clubfoot deformity has multi-segmental and multiplane characteristics. However, the majority of studies focused on the entire foot instead of separating the foot into different segments [24,27,31,32,35,36,39,40,41,42,43]. Notably, in recent studies, more frequently, a multi-segment foot model, such as the Oxford Foot Model, was used during the 3D gait analyses [25,26,28,29,30,33,34,37,38]. Although this resulted in an increased number of investigated kinematic parameters, combining a traditional model with a multi-segmental foot model does aid in fully grasping the complexity of the clubfoot deformity and treatment outcome [25,30,33,38,48]. A traditional single-segmental foot model is limited in representing foot motion in the frontal and transversal plane while considering the characteristics of the clubfoot foot motions, such as supination and adduction, are clinically highly relevant. Using a multi-segmental foot model allowed for a detailed analysis of hindfoot and forefoot motion [50], which resulted in the large number of differences at the foot level shown in the results. 

In order to assist with the interpretation of the numerous gait- and foot-specific kinematic parameters that are included in the traditional and multi-segmental models, gait and foot indices are used. Although the numerous kinematic parameters give detailed information regarding a child’s gait pattern, all these parameters can be difficult to interpret. Therefore, it could be preferred to use gait or foot indices, in which multiple kinematic parameters are combined into a single score, to assess the overall gait and foot quality in clinical practice [51,52,53]. These gait indices were implemented in several studies and showed that the overall gait and foot quality is different in clubfoot patients [28,31,33,36,39,43].

In ten of the twelve included studies that compare clubfoot without a relapse to healthy controls, one or more patients had received additional surgical treatment besides the initial casting and bracing phase of the Ponseti treatment, most likely because of a former relapse [25,27,32,36,38,39,40,42,43]. This could affect the kinematic results due to an increased variability among clubfoot patients within a study population since previous studies showed that surgical treatment, for example, can affect the ankle range of motion [45,54]. To better understand the occurrence of relapse and to evaluate the effect of relapse treatment, it is—from a clinical point of view—necessary to investigate successfully treated clubfeet without a relapse or additional surgical treatment and relapsed clubfeet separately.

Seven studies, including data from relapse patients prior to additional treatment [28,29,30,31,33,34,35], revealed multiple additional kinematic parameters on which relapse clubfoot patients differ from healthy controls. As such, gait analyses might play an important role in the early identification of relapse and determining the necessity of additional treatment, which could prevent the need for major surgical interventions [49,55,56,57]. In the future, the comparison of clubfoot with and without relapse will be necessary in order to optimize the Ponseti treatment and the detection of relapsed clubfoot. Furthermore, gait analyses can be used to evaluate the outcome of additional treatment for a relapse [11,45,58]. Recent studies investigating the effect of TATT and repeated Ponseti treatment already gave the first insight into kinematic changes after treatment [29,31,59]. Future studies should continue investigating the effect of treatment to aid in optimizing and developing additional (physio)therapy or surgical treatment.

The lack of a clear definition for a relapsed clubfoot was also apparent in the literature describing gait analyses [8]. Some authors used specific relapse treatment as an inclusion criterion for the relapse group, while others based this on planned treatment or an aberrant gait pattern [28,29,30,31,33,34,35]. Considering the heterogeneous nature of a relapse [52,55] and different purposes for applying gait analyses, composing a homogeneous relapse group will be challenging but is important for the comparison and interpretation of results. 

Besides the lack of a clear definition for a relapsed clubfoot, this review has a few other limitations. First of all, the quality of a systematic review depends highly on the number and the quality of the included studies. Of the presented kinematic parameters, only twelve could be included in a meta-analysis because of the diverse and numerous reported outcome measures. More homogeneity in measured kinematic variables should be taken into account in order to improve the comparison between separate studies. Secondly, all included studies compared children treated with the Ponseti method and healthy control children, but often the selection of participants and current status of the included patients was unclear, which could have led to selection bias. Thirdly, it seems that data from the same patients has been included in multiple studies. Furthermore, since bilateral club feet are highly correlated [60], future studies should show analyses of both sides if bilateral affected clubfoot patients are measured, especially if these are combined with data from unilateral affected clubfoot patients. However, we do believe that, as a strength of this review, the included studies describe a general population of clubfeet patients treated with the Ponseti method, and as such, the presented results are informative for the clinic. Moreover, the combination of meta-analyses and qualitative analyses led to a comprehensive overview of all studied kinematic characteristics. 

## 5. Conclusions

In conclusion, this systematic review showed that there are several differences in joint angles during gait in children with Ponseti-treated clubfoot with and without relapse compared to healthy controls. When comparing Ponseti-treated clubfoot children without relapse to healthy controls, deviations are mainly found in the sagittal and frontal plane ankle joint kinematics. When comparing children with pre-treatment relapsed clubfeet and healthy controls, deviations are found at foot level in all three planes and multiple phases of gait. We, therefore, emphasize the importance of evaluating the gait pattern of children with clubfoot during clinical follow-up. Being aware of gait impairments in treated clubfoot patients is useful for optimizing the Ponseti method, the detection of relapsed clubfoot, and developing additional (physio) therapy or surgery. However, the question remains as to what functional and/or long-term problems these gait impairments lead to and whether or not these problems could be addressed with additional treatment. Hence, from a clinical point of view, future studies should shift their focus to comparing clubfoot with and without relapse, evaluating the impact of gait impairments, for example, in terms of participation with peers, and investigating the effect of (additional) treatment. 

## Figures and Tables

**Table 1:** Study characteristics for each included study (only characteristics concerning Ponseti vs. controls are provided).

Study	Treatment	N (feet)	Gender	Mean Age in Years (Range) or ± SD	N TATT	N Additional Treatment	Marker Position	Dimeglio Scale ^1^
**Karol** **2009** [24]	Ponseti	- (34 feet)	-	5	^2^		-	12.8 (10–15) ^a^
	Control	- (17)	-	5				
**Church** **2012** [25]	Ponseti	22 (35 feet) ^3^	9M	6.3 ± 1.4 (5.0–10.0)	1 subject		Multi-segment foot model and single-segment marker set	4.0 (3.0) ^b^
	Control	34	-	- (4.0–17.0)				
**Duffy** **2012** [36]	Ponseti	29 (42 feet)	20M	6.5 (5.0–8.0)	14 feet	4 subjects	-	-
	Control	26 (50 feet)	17M	7.9 (5.2–10.8)				
**Smith** **2014** [37]	Ponseti	18 (29 feet)	9M	29.2 ± 5.6	10 feet	6 feet	Milwaukee foot model	-
	Control	48	29M	23.2 ± 2.4				
**Mindler** **2014** [38]	Ponseti	32 (50 feet)	22M	6.0 (3.0–8.0)	5 feet		Cleveland model and Oxford foot model	-
	Control	15 (30 feet)	9M	6.0 (3.0–9.0)				
**Manousaki** **2016** [39]	Ponseti	20 (30 feet)	17M	7 ± 3.4 months	3 feet	3 feet	Plug in gait model including seven markers on the torso	11 (9–13) ^c^
	Control	16		8.5 (6.1–12) ^4^				
**Lööf** **2016** [40]	Ponseti	59 (89 feet)	41M	5.4 ± 0.5	3 feet		Plug in gait model	16 moderated, 48 severed, 24 very severed ^d^
	Control	28 (56 feet)	18M	5.5 ± 0.6				
**Jeans** **2018** [41]	Ponseti	50 (75 feet)	-	10			Plug in gait model	13.4 ± 1.9
	Control	20 (40 feet)	-	10				
**Manousaki 2019** [42]	Ponseti	20 (20 feet)	17M	7 ± 3.4 months		3 feet	Plug in gait model	-
	Control	16 (32 feet)		8.5 (6.1–12.0) ^4^				
**Loof** **2019** [43]	Ponseti	47 (69 feet)	35M	5.4 (0.5)	3 feet		Plug in gait model	15 moderated, 36 severed 17 very severed ^d^
	Control	28 (56 feet)	18M	5.5 (0.6)				
**Dussa** **2020** [26]	Ponseti overcorrected	14	-	9.9 (1.5)			Plug in gait model and Oxford foot model	-
	Control	25	-	9.9 (2.7)				
**Ferrando** **2020** [27]	Ponseti Control	22 (34 feet) 25 (50 feet)	14M 18M	8 ± 1 9 ± 2	11 feet	5 feet	-	-
**McCahill** **2020** [28]	Ponseti relapse	31	24M	8.3 (5–16)	10 subjects		Oxford foot model	-
	Control	30	21M	10.7 (5–16)				
**Mindler** **2020** [29]	Ponseti relapse	17 (25 feet)	11M	6.8 (5.1–9.1)			Cleveland model and Oxford foot model	-
	Control	18 (36 feet)	6M	6 (4–9)				
**Grin** **2021** [30]	Ponseti	11	9M	5.6 ± 1.6			Extended Helen Hayes and Oxford foot model	-
	Ponseti relapse	11	8M	5.7 ± 1.5				
	Control	15	8M	5.7 ± 1.4				
**Li** **2021** [31]	Ponseti relapse	17 (24 feet)	12M	6.34 ± 1.65 (4.47–10.2)			Helen Hayes model	-
	Control	16	M:F = 1.14:1	7.12 ± 2.23				
**Recordon** **2021** [32]	Ponseti	16 (23 feet)	-	15 (13–17)	^5^	^5^	-	5.8 ± 1.7
	Control	39 (78 feet)	-	Age-matched				
**Brierty** **2022** [35]	Ponseti relapse	16 (23 feet)	13M	5.58 (3.27–8.57)			Plug in gait model and Oxford foot model	-
	Control	9	-	6.31 (4.47–7.96)				
**Grin** **2022** [33]	Ponseti	18	18M	5.39 ± 1.46			Extended Helen Hayes and Oxford foot model	-
	Ponseti relapse	13	8M	5.46 ± 1.51				
	Control	21	12M	6 ± 1.57				
**Wijnands** **2022** [34]	Ponseti	15	12M	5.13 ± 1.25			Extended Helen Hayes and Oxford foot model	-
	Ponseti relapse	10	6M	5.70 ± 1.57				
	Control	19	11M	5.79 ± 1.40				

^1^ Dimeglio scale: classification on a scale of 0–20 based on eight items, divided into four grades (benign, moderate, severe, very severe) [44], ^2^ included children with a tibialis anterior tendon transfer (TATT) as part of the Ponseti method but did not report the number of feet included. ^3^ multi-segment foot model data are only available for 23 of 35 involved feet. ^4^ median instead of mean. ^5^ included eight subjects with additional treatment but did not report which treatment. ^a^ mean (range). ^b^ median (interquartile distance). ^c^ medial (range). ^d^ number of feet with a moderate (5–10), severe (11–15), or very severe (16–20) score on the Dimeglio scale, a total of 88 feet has been scaled in Lööf 2016 and 68 feet in Lööf 2019. - no information was provided.

**Table 2:** Clubfoot versus controls—Outcome measures included the qualitative analysis presenting significant differences. Parameters without significant differences are presented in Table A3.

Outcome Measure		Moment in Gait Cycle	Studies	Significance
Foot	Mean tibial torsion (EXT) Foot progression (EXT)	Stance Preswing	[25] [37]	Clubfoot < controls Clubfoot > controls
Forefoot vs. hindfoot	ROM sagittal (DF/PF) Plantarflexion	Gait cycle 20% gait cycle ^1^	[30,38] [37]	Conflicting outcome ^2^ Clubfoot < controls
Forefoot vs. *tibia*	ROM sagittal (DF/PF) ROM frontal (PRO/SUP) ROM transversal (AB/AD)	Gait cycle Gait cycle Gait cycle	[30,38] [30,38] [30,38]	Conflicting outcome ^2^ Conflicting outcome ^2^ Conflicting outcome ^2^
Ankle	Dorsiflexion Max. plantarflexion Dorsiflexion	Mid-stance Terminal stance Swing ^1^	[24,39] [40] [30,37]	Conflicting outcome ^2^ Clubfoot < controls Conflicting outcome ^2^
Knee	Max. extension Max. extension Max. flexion	Mid-stance 2nd half of stance Swing	[36] [38] [30,38]	Clubfoot < controls Clubfoot < controls Conflicting outcome ^2^
Hip	Mean abduction Max. rotation (EXT) Mean rotation (EXT)	Gait cycle Gait cycle Mid-stance	[38] [30,38] [36]	Clubfoot > controls Conflicting outcome ^2^ Clubfoot > controls
Total gait scores	GDI	Gait cycle	[36,39,43]	Clubfoot < controls

Abbreviations: ROM = range of motion/PF = plantarflexion/DF = dorsiflexion/INT = internal rotation/EXT = external rotation/AB = abduction/AD = adduction/PRO = pronation/SUP = supination Max. = maximum/GDI = gait deviation index. ^1^ information gained from figure. ^2^ in case of conflicting outcomes, additional information is provided in the text.

**Table 3:** Pre-treatment relapsed clubfoot vs. Controls—Outcome measures included the qualitative analysis presenting significant differences. Parameters without significant differences are presented in Table A4.

Outcome Measure		Moment in Gait Cycle	Studies	Significance
Foot	Shank-based foot rotation (INT) Foot progression angle (EXT) Foot progression angle (EXT) Shank-based foot rotation (INT)	Stance Stance 70% gait cycle ^1^ Swing	[30] [29] [35] [30]	Relapse > controls Relapse < controls Relapse < controls Relapse > controls
Hindfoot vs. *tibia*	Mean adduction ROM sagittal (DF/PF) ROM transversal (INT/EXT) Inversion Adduction Dorsiflexion Max. dorsiflexion Mean adduction	Gait cycle Gait cycle Gait cycle Gait cycle ^1^ Gait cycle ^1^ Initial contact Stance Stance	[28] [28,29,30] [28,29,30] [35] [35] [29,30] [29,30] [29,30]	Relapse > controls Conflicting outcome ^2^ Conflicting outcome ^2^ Relapse > controls Relapse > controls Conflicting outcome ^2^ Conflicting outcome ^2^ Conflicting outcome ^2^
Forefoot vs. hindfoot	ROM sagittal (DF/PF) ROM frontal (PRO/SUP) Max. plantarflexion Adduction Dorsiflexion Max. dorsiflexion Mean adduction Adduction Max. dorsiflexion Supination	Gait cycle Gait cycle Gait cycle Gait cycle^1^ Initial contact Stance Stance Toe-off Swing 80% gait cycle	[28,29,30] [28,29,30] [29,30] [35] [29,30] [29,30] [29,30] [30] [29,30] [29]	Conflicting outcome ^2^ Conflicting outcome ^2^ Conflicting outcome ^2^ Relapse > controls Conflicting outcome ^2^ Conflicting outcome ^2^ Conflicting outcome ^2^ Relapse > controls Conflicting outcome ^2^ Relapse > controls
Forefoot vs. *tibia*	ROM sagittal (DF/PF) ROM transversal (AB/AD) Max. plantarflexion Mean adduction Adduction Supination Mean adduction Plantarflexion Mean adduction Mean supination/pronation Dorsiflexion Adduction	Gait cycle Gait cycle Gait cycle Gait cycle Initial contact Initial contact Stance Toe-off Swing Swing 80% gait cycle 80% gait cycle	[29,30] [29,30] [29,30] [28] [29] [29] [29,30] [30] [29,30] [29,30] [29] [29]	Conflicting outcome ^2^ Conflicting outcome ^2^ Conflicting outcome ^2^ Relapse > controls Relapse > controls Relapse > controls Relapse > controls Relapse < controls Conflicting outcome ^2^ Conflicting outcome ^2^ Relapse > controls Relapse > controls
Ankle	ROM sagittal (PF/DF) Max. dorsiflexion Plantarflexion	Gait cycle Gait cycle Toe-off	[29,30] [29,34] [30]	Relapse < controls Conflicting outcome ^2^ Relapse < controls
Knee	Mean rotation (EXT) Flexion	Stance End of swing ^1^	[29] [35]	Relapse < controls Relapse > control
Hip	Mean rotation (INT) External rotation Abduction	Stance 30–60% gait cycle ^1^ 50–90% gait cycle ^1^	[29,30] [35] [35]	Conflicting outcome ^2^ Relapse > controls Relapse > controls
Total gait scores	GDI GDI* cFDI* Foot profile score FVS hindfoot sagittal FVS hindfoot frontal FVS hindfoot transversal FVS forefoot sagittal FVS forefoot transversal	Gait cycle Gait cycle Gait cycle Gait cycle Gait cycle Gait cycle Gait cycle Gait cycle Gait cycle	[31] [33] [33] [28] [28] [28] [28] [28] [28]	Deviated from normal ^3^ Deviated from normal ^3^ Deviated from normal ^3^ Relapse > controls Relapse > controls Relapse > controls Relapse > controls Relapse > controls Relapse > controls

Abbreviations: ROM = range of motion/PF = plantarflexion/DF = dorsiflexion/INT = internal rotation/EXT = external rotation/AB = abduction/AD = adduction/PRO = pronation/SUP = supination Max. = maximum/GDI = gait deviation index/GDI* = scaled gait deviation index/cFDI* = clubfoot deviation index/FVS = foot variable score. ^1^ information gained from figure. ^2^ in case of conflicting outcomes, additional information is provided in the text. ^3^ a score below 90 means a deviated gait pattern compared to controls [42]. Only significant results are included in this table.

## Data Availability

Data sharing not applicable.

## Appendix A. Example Literature Search Embase.com

**Embase.com**

(clubfoot/de OR ‘pes equinovarus’/exp OR (clubfoot OR clubfeet OR club-foot OR club-feet OR talipes OR equinovarus OR equino-varus ):ab,ti) AND (therapy/exp OR ‘treatment outcome’/exp OR surgery/exp OR therapy:lnk OR surgery:lnk OR ‘clinical trial’/exp OR relapse/exp OR ‘follow up’/exp OR ‘evaluation study’/exp OR rehabilitation/exp OR rehabilitation:lnk OR ‘single blind procedure’/exp OR ‘double blind procedure’/exp OR ‘triple blind procedure’/exp OR (surg* OR therap* OR treat* OR ponseti OR cast* OR outcome* OR nonoperat* OR nonsurg* OR comprehensive* OR release* OR interven* OR management* OR conservative* OR trial* OR random* OR correct* OR relaps* OR recur* OR (follow* NEXT/1 up*) OR followup* OR evaluat* OR rehabilitat* OR ((double OR single OR triple) NEXT/1 (blind* OR mask*)) OR Physiotherap*):ab,ti) AND (gait/exp OR ‘gait disorder’/exp OR electromyogram/exp OR biomechanics/exp OR ‘pressure measurement’/de OR (gait OR ((force OR forces OR pressure*) NEAR/3 (distribut* OR peak OR foot OR measur* OR plantar*)) OR EMG OR pedobarograph* OR electromyogr* OR Biomechanic*)).

## Appendix B. Risk of Bias

**Table A1:** Risk of bias for each included study.

Study	Selection	Groups	Measurement	Blinded	Prognostic Factors
** Karol 2009 ** [24]	−	+	+	?	+
** Church 2012 ** [25]	+	?	+	?	?
** Duffy 2012 ** [36]	?	+	+	?	?
** Smith 2014 ** [37]	−	−	+	−	−
** Mindler 2014 ** [38]	?	+	+	?	?
** Manousaki 2016 ** [39]	+	?	+	?	?
** Lööf 2016 ** [40]	?	+	+	?	?
** Jeans 2018 ** [41]	−	?	+	?	+
** Manousaki 2019 ** [42]	+	?	+	?	−
** Lööf 2019 ** [43]	?	+	+	?	+
** Dussa 2020 ** [26]	+	+	+	?	?
** Ferrando 2020 ** [27]	+	+	+	?	?
** McCahill 2020 ** [28]	?	+	+	?	?
** Mindler 2020 ** [29]	+	+	+	?	?
** Grin 2021 ** [30]	−	+	+	?	?
** Li 2021 ** [31]	+	+	+	?	+
** Recordon 2021 ** [32]	?	?	+	?	+
** Brierty 2022 ** [35]	?	+	+	?	?
** Grin 2022 ** [33]	−	+	+	?	?
** Wijnands 2022 ** [34]	−	+	+	?	?

**Selection:** if stated “all patients in period…” or “consecutive patients”; and thus no selection has been made +. **Groups:** if the different groups are clearly defined and comparable with each other (e.g., based on age). **Measurement:** if a valid measurement system/gait analysis system was used. **Blinded:** if outcome measurements were independently/blindly determined. **Prognostic factors:** if clubfoot initial and current classification of clubfeet patients have been described, and the description of the control group states healthy controls. + Low risk/− High risk/? Unclear.

## Appendix C. Results Dussa et al. (Overcorrected Clubfoot vs. Controls)

**Table A2:** Results overcorrected Ponseti clubfoot vs. controls (Dussa et al) [24].

Outcome Measure		Moment in Gait Cycle	Significance
Hindfoot vs. *tibia*	Peak dorsiflexion Peak eversion Peak internal rotation ROM sagittal (DF/PF) ROM frontal (INV/EV) ROM transversal (INT/EXT) Plantar flexion Inversion	Stance Stance Stance Stance Stance Stance Toe-off Toe-off	Overcorrect < controls Overcorrect > controls No significance Overcorrect < controls Overcorrect < controls Overcorrect > controls No significance Overcorrect < controls
Forefoot vs. hindfoot	Mean supination Peak dorsiflexion Peak pronation Peak adduction Sagittal ROM (PF/DF) Frontal ROM (PRO/SUP) Transversal ROM (AB/AD)	Gait cycle Stance Stance Stance Stance Stance Stance	Overcorrect > controls Overcorrect > controls Overcorrect > controls No significance No significance No significance No significance
Hallux vs. forefoot	Sagittal ROM (FLEX/EXT) Flexion Mean flexion Sagittal ROM (FLEX/EXT)	Stance Toe-off Swing Swing	No significance Overcorrect < controls Overcorrect < controls No significance

## Appendix D. Ponseti vs. Controls—Additional Outcome Measures Presented in Different Studies

**Table A3:** Outcome measures included the qualitative analysis with no significant differences.

Outcome Measure		Moment in Gait Cycle	Studies	Significance
** Foot **	Foot progression Shank-based foot rotation (INT)	Mid-stance Swing	[32] [30]	No significant difference No significant difference
** Hindfoot vs. *tibia***	ROM sagittal (DF/PF) ROM transversal (INT/EXT) Max. plantar flexion Dorsiflexion Max. dorsiflexion Mean dorsiflexion Mean adduction Mean inversion/eversion Plantar flexion Adduction Mean adduction Mean plantar/dorsiflexion Max. dorsiflexion Mean inversion/eversion	Gait cycle Gait cycle Gait cycle Initial contact Stance Stance Stance Stance Toe-off Toe-off Swing Swing Swing Swing	[30,38] [30,38] [30] [30] [30] [30] [25,30] [30] [30] [30] [30] [30] [30] [30]	No significant difference No significant difference No significant difference No significant difference No significant difference No significant difference No significant difference No significant difference No significant difference No significant difference No significant difference No significant difference No significant difference No significant difference
** Forefoot vs. hindfoot **	ROM frontal (PRO/SUP) ROM transversal (AB/AD) Max. plantar flexion Dorsiflexion Max. dorsiflexion Mean dorsiflexion Mean adduction Mean supination/pronation Plantar flexion Adduction Mean adduction Mean plantar/dorsiflexion Max. dorsiflexion Mean supiation/pronation	Gait cycle Gait cycle Gait cycle Initial contact Stance Stance Stance Stance Toe-off Toe-off Swing Swing Swing Swing	[30,38] [30,38] [30] [30] [30] [30] [30] [25,30] [30] [30] [30] [30] [30] [30]	No significant difference No significant difference No significant difference No significant difference No significant difference No significant difference No significant difference No significant difference No significant difference No significant difference No significant difference No significant difference No significant difference No significant difference
** Forefoot vs. *tibia***	Peak plantar flexion Dorsiflexion Max. dorsiflexion Mean dorsiflexion Mean adduction Mean supination/pronation Plantar flexion Adduction Mean adduction Mean plantar/dorsiflexion Max. dorsiflexion Mean supination/pronation	Gait cycle Initial contact Stance Stance Stance Stance Toe-off Toe-off Swing Swing Swing Swing	[30] [30] [30] [30] [30] [30] [30] [30] [30] [30] [30] [30]	No significant difference No significant difference No significant difference No significant difference No significant difference No significant difference No significant difference No significant difference No significant difference No significant difference No significant difference No significant difference
** Ankle **	Mean dorsiflexion ROM PF/DF Dorsiflexion Mean dorsiflexion	Stance Stance End of swing Terminal swing	[24,30] [32] [24,39] [40]	No significant difference No significant difference No significant difference No significant difference
** Knee **	Mean rotation ROM sagittal	Gait cycle Gait cycle	[38] [30]	No significant difference No significant difference
** Hip **	External rotation	Initial contact	[40]	No significant difference
**Pelvis**	Mean tilt ROM transversal Max. rotation (EXT) Max. rotation (INT)	Gait cycle Gait cycle Gait cycle Gait cycle	[30] [30] [30] [30]	No significant difference No significant difference No significant difference No significant difference
** Total gait scores **	GPS overall GPS affected side GVS pelvis anterior/posterior GVS pelvis int/ext rotation GVS pelvis up/down GVS hip flexion/extension GVS hip adduction/abduction GVS hip int/ext rotation GVS knee flexion/extension GVS ankle dorsal/plantar flexion GVS foot int/ext rotation GDI * FDI * cFDI *	Gait cycle Gait cycle Gait cycle Gait cycle Gait cycle Gait cycle Gait cycle Gait cycle Gait cycle Gait cycle Gait cycle Gait cycle Gait cycle Gait cycle	[42] [42] [42] [42] [42] [42] [42] [42] [42] [42] [42] [33] [33] [33]	- - - - - - - - - - - No deviation ^1^ No deviation ^1^ No deviation ^1^

Abbreviations: ROM = range of motion/PF = plantarflexion/DF = dorsiflexion/INT = internal rotation/EXT = external rotation/AB = abduction/AD = adduction/PRO = pronation/SUP = supination Max. = maximum/GDI = gait deviation index/GDI * = scaled gait deviation index/ FDI* = scaled foot deviation index/cFDI * = clubfoot deviation index/ ^1^ a score below 90 means a deviated gait pattern compared to controls [42], ‘-’ outcome compared with controls, but no statistical information was provided. All parameters: Clubfoot > controls.

## Appendix E. Relapsed Clubfoot Pre-Treatment vs. Controls—Additional Outcome Measures Presented in Different Studies

**Table A4:** Outcome measures included qualitative analysis with no significant differences.

Outcome Measure		Moment in Gait Cycle	Studies	Significance
** Foot **	Foot progression angle	Gait cycle	[28]	Foot progression angle
** Hindfoot vs. *tibia***	ROM frontal (INV/EV) Max. plantarflexion Mean dorsiflexion Mean inversion Inversion/eversion Adduction Mean dorsiflexion Mean inversion/eversion Plantarflexion Adduction Mean adduction Mean plantar/dorsiflexion Max. dorsiflexion Mean inversion/eversion Varus	Gait cycle Gait cycle Gait cycle Gait cycle Initial contact Initial contact Stance Stance Toe-off Toe-off Swing Swing Swing Swing 80% gait cycle	[26,27,28] [27,28] [26] [26] [27] [27] [28] [28] [28] [28] [27,28] [28] [28] [28] [27]	No significant difference No significant difference No significant difference No significant difference No significant difference No significant difference No significant difference No significant difference No significant difference No significant difference No significant difference No significant difference No significant difference No significant difference No significant difference
** Forefoot vs. hindfoot **	ROM transversal (AB/AD) Mean dorsiflexion Mean supination Mean adduction Supination Adduction Mean dorsiflexion Mean supination/pronation Plantarflexion Mean adduction Mean plantar/dorsiflexion Mean supination/pronation Supination	Gait cycle Gait cycle Gait cycle Gait cycle Initial contact Initial contact Stance Stance Toe-off Swing Swing Swing 80% gait cycle	[26,27,28] [26] [26] [26] [27] [27] [28] [28] [28] [27,28] [28] [28] [27]	No significant difference No significant difference No significant difference No significant difference No significant difference No significant difference No significant difference No significant difference No significant difference No significant difference No significant difference No significant difference No significant difference
** Forefoot vs. *tibia***	ROM frontal (PRO/SUP) Mean dorsiflexion Mean supination Dorsiflexion Max. dorsiflexion Mean dorsiflexion Mean supination/pronation Adduction Mean plantar/dorsiflexion Max. dorsiflexion	Gait cycle Gait cycle Gait cycle Initial contact Stance Stance Stance Toe-off Swing Swing	[28] [26] [26] [27,28] [27,28] [28] [28] [28] [28] [28]	No significant difference No significant difference No significant difference No significant difference No significant difference No significant difference No significant difference No significant difference No significant difference No significant difference
** Ankle **	Max. plantarflexion Dorsiflexion Mean dorsiflexion Max. dorsiflexion Max. dorsiflexion Mean plantar/dorsiflexion	Gait cycle Initial contact Stance Stance Swing Swing	[28] [28] [28] [28] [28] [28]	No significant difference No significant difference No significant difference No significant difference No significant difference No significant difference
** Knee **	ROM sagittal (PF/DF) Max. extension Max. flexion	Gait cycle Stance Swing	[28] [28] [28]	No significant difference No significant difference No significant difference
** Hip **	Max. rotation (EXT)	Gait cycle	[28]	Max. rotation (EXT)
**Pelvis**	Mean tilt ROM transversal Max. rotation (EXT) Max. rotation (INT)	Gait cycle Gait cycle Gait cycle Gait cycle	[28] [28] [28] [28]	No significant difference No significant difference No significant difference No significant difference
** Total gait scores **	FDI * FVS forefoot frontal	Gait cycle Gait cycle	[31] [26]	No deviation ^1^ No significant difference

Abbreviations: ROM = range of motion/PF = plantarflexion/DF = dorsiflexion/INT = internal rotation/EXT = external rotation/AB = abduction/AD = adduction/PRO = pronation/SUP = supination/Max. = maximum/* = scaled foot deviation index/FVS = foot variable score. ^1^ a score below 90 means a deviated gait pattern compared to controls [42].

## References

[1] Mustari M.N., Faruk M., Bausat A., Fikry A. (2022). Congenital Talipes Equinovarus: A Literature Review. Ann. Med. Surg..

[2] Dibello D., Torelli L., Di Carlo V., D’Adamo A.P., Faletra F., Mangogna A., Colin G. (2022). Incidence of Congenital Clubfoot: Preliminary Data from Italian CeDAP Registry. Int. J. Environ. Res. Public Health.

[3] Esbjörnsson A.-C., Johansson A., Andriesse H., Wallander H. (2021). Epidemiology of Clubfoot in Sweden from 2016 to 2019: A National Register Study. PLoS ONE.

[4] Ponseti I.V., Zhivkov M., Davis N., Sinclair M., Dobbs M.B., Morcuende J.A. (2006). Treatment of the Complex Idiopathic Clubfoot. Clin. Orthop. Relat. Res..

[5] Gray K., Pacey V., Gibbons P., Little D., Burns J. (2014). Interventions for Congenital Talipes Equinovarus (Clubfoot). Cochrane Database Syst. Rev..

[6] Bergerault F., Fournier J., Bonnard C. (2013). Idiopathic Congenital Clubfoot: Initial Treatment. Orthop. Traumatol. Surg. Res..

[7] Shabtai L., Specht S.C., Herzenberg J.E. (2014). Worldwide Spread of the Ponseti Method for Clubfoot. World J. Orthop..

[8] Thomas H.M., Sangiorgio S.N., Ebramzadeh E., Zionts L.E. (2019). Relapse Rates in Patients with Clubfoot Treated Using the Ponseti Method Increase with Time: A Systematic Review. JBJS Rev..

[9] Gelfer Y., Wientroub S., Hughes K., Fontalis A., Eastwood D.M. (2019). Congenital Talipes Equinovarus: A Systematic Review of Relapse as a Primary Outcome of the Ponseti Method. Bone Jt. J..

[10] Hu W., Ke B., Niansu X., Li S., Li C., Lai X., Huang X. (2022). Factors Associated with the Relapse in Ponseti Treated Congenital Clubfoot. BMC Musculoskelet. Disord..

[11] Radler C. (2021). The Treatment of Recurrent Congenital Clubfoot. Foot Ankle Clin..

[12] Gaber K., Mir B., Shehab M., Kishta W. (2022). Updates in the Surgical Management of Recurrent Clubfoot Deformity: A Scoping Review. Curr. Rev. Musculoskelet. Med..

[13] WHO-FIC CC (2018). International Classification of Functioning, Disability and Health, Children & Youth Version.

[14] Gelfer Y., Leo D.G., Russell A., Bridgens A., Perry D.C., Eastwood D.M. (2022). The Outcomes of Idiopathic Congenital Talipes Equinovarus: A Core Outcome Set for Research and Treatment. Bone Jt. Open.

[15] Cimolin V., Galli M. (2014). Summary Measures for Clinical Gait Analysis: A Literature Review. Gait Posture.

[16] Graf A., Wu K.W., Smith P.A., Kuo K.N., Krzak J., Harris G. (2012). Comprehensive Review of the Functional Outcome Evaluation of Clubfoot Treatment: A Preferred Methodology. J. Pediatr. Orthop. B.

[17] Karol L.A., Jeans K.A. (2011). Assessment of Clubfoot Treatment Using Movement Analysis. J. Exp. Clin. Med..

[18] Bent M., Hauschild M., Rethlefsen S.A., Wren T.A.L., Liang A., Goldstein R.Y., Kay R.M. (2023). Gait Analysis Characteristics in Relapsed Clubfoot. J. Pediatr. Orthop..

[19] Tuinsma A.B.M., Vanwanseele B., van Oorschot L., Kars H.J.J., Grin L., Reijman M., Besselaar A.T., van der Steen M.C. (2018). Gait Kinetics in Children with Clubfeet Treated Surgically or with the Ponseti Method: A Meta-Analysis. Gait Posture.

[20] Pierz K.A., Lloyd J.R., Solomito M.J., Mack P., Õunpuu S. (2020). Lower Extremity Characteristics in Recurrent Clubfoot: Clinical and Gait Analysis Findings That May Influence Decisions for Additional Surgery. Gait Posture.

[21] Liberati A., Altman D.G., Tetzlaff J., Mulrow C., Gotzsche P.C., Ioannidis J.P., Clarke M., Devereaux P.J., Kleijnen J., Moher D. (2009). The PRISMA Statement for Reporting Systematic Reviews and Meta-Analyses of Studies That Evaluate Health Care Interventions: Explanation and Elaboration. PLoS Med..

[22] Moher D., Liberati A., Tetzlaff J., Altman D.G., Group P. (2009). Preferred Reporting Items for Systematic Reviews and Meta-Analyses: The PRISMA Statement. BMJ.

[23] Page M.J., McKenzie J.E., Bossuyt P.M., Boutron I., Hoffmann T.C., Mulrow C.D., Shamseer L., Tetzlaff J.M., Akl E.A., Brennan S.E. (2021). The PRISMA 2020 Statement: An Updated Guideline for Reporting Systematic Reviews. BMJ.

[24] Karol L.A., Jeans K., Elhawary R. (2009). Gait Analysis after Initial Nonoperative Treatment for Clubfeet: Intermediate Term Followup at Age 5. Clin. Orthop. Relat. Res..

[25] Church C., Coplan J.A., Poljak D., Thabet A.M., Kowtharapu D., Lennon N., Marchesi S., Henley J., Starr R., Mason D. (2012). A Comprehensive Outcome Comparison of Surgical and Ponseti Clubfoot Treatments with Reference to Pediatric Norms. J. Child. Orthop..

[26] Dussa C.U., Böhm H., Döderlein L., Forst R., Fujak A. (2020). Does an Overcorrected Clubfoot Caused by Surgery or by the Ponseti Method Behave Differently?. Gait Posture.

[27] Ferrando A., Salom M., Page A., Perez-Girbes A., Atienza C., Minguez M.F., Prat J. (2020). Talipes Equinovarus Treatment in Infants Treated by the Ponseti Method Compared with Posterior-Only Release: A Mid-Childhood Comparison of Results. J. Foot Ankle Surg..

[28] McCahill J.L., Stebbins J., Harlaar J., Prescott R., Theologis T., Lavy C. (2020). Foot Function during Gait and Parental Perceived Outcome in Older Children with Symptomatic Club Foot Deformity. Bone Jt. Open.

[29] Mindler G.T., Kranzl A., Radler C. (2020). Normalization of Forefoot Supination after Tibialis Anterior Tendon Transfer for Dynamic Clubfoot Recurrence. J. Pediatr. Orthop..

[30] Grin L., van der Steen M.C., Wijnands S.D.N., van Oorschot L., Besselaar A.T., Vanwanseele B. (2021). Forefoot Adduction and Forefoot Supination as Kinematic Indicators of Relapse Clubfoot. Gait Posture.

[31] Li J., Xun F., Li Y., Liu Y., Xu H., Canavese F. (2022). Three-Dimensional Gait Analysis in Children with Recurrent Idiopathic Clubfoot Undergoing Complete Tibialis Anterior Tendon Transfer. J. Pediatr. Orthop. B.

[32] Recordon J.A.F., Halanski M.A., Boocock M.G., McNair P.J., Stott N.S., Crawford H.A. (2021). A Prospective, Median 15-Year Comparison of Ponseti Casting and Surgical Treatment of Clubfoot. J. Bone Jt. Surg. Am..

[33] Grin L., Wijnands S., Besselaar A., van Oorschot L., Vanwanseele B., van der Steen M. (2022). The Relation between Clinical and Objective Gait Scores in Clubfoot Patients with and without a Relapse. Gait Posture.

[34] Wijnands S.D.N., van der Steen M.C., Grin L., van Oorschot L., Besselaar A.T., Vanwanseele B. (2022). Muscle-Tendon Properties and Functional Gait Outcomes in Clubfoot Patients with and without a Relapse Compared to Typically Developing Children. Gait Posture.

[35] Brierty A., Horan S., Giacomozzi C., Johnson L., Bade D., Carty C.P. (2022). Kinematic Differences in the Presentation of Recurrent Congenital Talipes Equinovarus (Clubfoot). Gait Posture.

[36] Duffy C.M., Salazar J.J., Humphreys L., McDowell B.C. (2013). Surgical versus Ponseti Approach for the Management of CTEV: A Comparative Study. J. Pediatr. Orthop..

[37] Smith P.A., Kuo K.N., Graf A.N., Krzak J., Flanagan A., Hassani S., Caudill A.K., Dietz F.R., Morcuende J., Harris G.F. (2014). Long-Term Results of Comprehensive Clubfoot Release versus the Ponseti Method: Which Is Better?. Clin. Orthop. Relat. Res..

[38] Mindler G.T., Kranzl A., Lipkowski C.A.M., Ganger R., Radler C. (2014). Results of Gait Analysis Including the Oxford Foot Model in Children with Clubfoot Treated with the Ponseti Method. J. Bone Jt. Surg..

[39] Manousaki E., Czuba T., Hägglund G., Mattsson L., Andriesse H. (2016). Evaluation of Gait, Relapse and Compliance in Clubfoot Treatment with Custom-Made Orthoses. Gait Posture.

[40] Lööf E., Andriesse H., André M., Böhm S., Broström E.W. (2016). Gait in 5-Year-Old Children with Idiopathic Clubfoot: A Cohort Study of 59 Children, Focusing on Foot Involvement and the Contralateral Foot. Acta Orthop..

[41] Jeans K.A., Karol L.A., Erdman A.L., Stevens W.R. (2018). Functional Outcomes Following Treatment for Clubfoot Ten-Year Follow-Up. J. Bone Jt. Surg..

[42] Manousaki E., Esbjörnsson A.C., Mattsson L., Andriesse H. (2019). Correlations between the Gait Profile Score and Standard Clinical Outcome Measures in Children with Idiopathic Clubfoot. Gait Posture.

[43] Lööf E., Andriesse H., André M., Böhm S., Iversen M.D., Broström E.W. (2019). Gross Motor Skills in Children with Idiopathic Clubfoot and the Association between Gross Motor Skills, Foot Involvement, Gait, and Foot Motion. J. Pediatr. Orthop..

[44] Dimeglio A., Bensahel H., Souchet P., Mazeau P., Bonnet F. (1995). Classification of Clubfoot. J. Pediatr. Orthop. B.

[45] Karol L.A., Jeans K.A. (2021). This Is a Narrative Review of the Functional Evaluation of Clubfoot Treatment with Gait Analysis. Ann. Transl. Med..

[46] Perry J., Burnfield J.M., SLACK Incorporated (2010). Gait Analysis, Normal and Pathological Function.

[47] Gintautienė J., Čekanauskas E., Barauskas V., Žalinkevičius R. (2016). Comparison of the Ponseti Method versus Early Tibialis Anterior Tendon Transfer for Idiopathic Clubfoot: A Prospective Randomized Study. Medicina.

[48] Theologis T.N., Harrington M.E., Thompson N., Benson M.K.D. (2003). Dynamic Foot Movement in Children Treated for Congenital Talipes Equinovarus. J. Bone Jt. Surg..

[49] Sankar W.N., Rethlefsen S.A., Weiss J., Kay R.M. (2009). The Recurrent Clubfoot: Can Gait Analysis Help Us Make Better Preoperative Decisions?. Clin. Orthop. Relat. Res..

[50] McCahill J., Stebbins J., Koning B., Harlaar J., Theologis T. (2018). Repeatability of the Oxford Foot Model in Children with Foot Deformity. Gait Posture.

[51] Baker R., McGinley J.L., Schwartz M.H., Beynon S., Rozumalski A., Graham H.K., Tirosh O. (2009). The Gait Profile Score and Movement Analysis Profile. Gait Posture.

[52] Schwartz M.H., Rozumalski A. (2008). The Gait Deviation Index: A New Comprehensive Index of Gait Pathology. Gait Posture.

[53] McCahill J., Stebbins J., Lewis A., Prescott R., Harlaar J., Theologis T. (2019). Validation of the Foot Profile Score. Gait Posture.

[54] Dobbs M.B., Nunley R., Schoenecker P.L. (2006). Long-Term Follow-up of Patients with Clubfeet Treated with Extensive Soft-Tissue Release. J. Bone Jt. Surg..

[55] Stouten J.H., Besselaar A.T., Van Der Steen M.C. (2018). Identification and Treatment of Residual and Relapsed Idiopathic Clubfoot in 88 Children. Acta Orthop..

[56] Ponseti I.V. (2002). Relapsing Clubfoot: Causes, Prevention, and Treatment. Iowa Orthop. J..

[57] Dietz F.R. (2006). Treatment of a Recurrent Clubfoot Deformity after Initial Correction with the Ponseti Technique. Instr. Course Lect..

[58] Jeans K.A., Erdman A.L., Jo C.-H.H., Karol L.A. (2016). A Longitudinal Review of Gait Following Treatment for Idiopathic Clubfoot: Gait Analysis at 2 and 5 Years of Age. J. Pediatr. Orthop..

[59] Liu Y.-B., Jiang S.-Y., Zhao L., Yu Y., Zhao D.-H. (2020). Can Repeated Ponseti Management for Relapsed Clubfeet Produce the Outcome Comparable with the Case Without Relapse? A Clinical Study in Term of Gait Analysis. J. Pediatr. Orthop..

[60] Gray K., Gibbons P., Little D., Burns J. (2014). Bilateral Clubfeet Are Highly Correlated: A Cautionary Tale for Researchers. Clin. Orthop. Relat. Res..

